# Visual Summary Identification From Scientific Publications *via* Self-Supervised Learning

**DOI:** 10.3389/frma.2021.719004

**Published:** 2021-08-19

**Authors:** Shintaro Yamamoto, Anne Lauscher, Simone Paolo Ponzetto, Goran Glavaš, Shigeo Morishima

**Affiliations:** ^1^ Department of Pure and Applied Physics, Waseda University, Tokyo, Japan; ^2^ Data and Web Science Group, University of Mannheim, Mannheim, Germany; ^3^ Waseda Research Institute for Science and Engineering, Tokyo, Japan

**Keywords:** scientific publication mining, multimodal retrieval, visual summary identification, scientific figure, document analysis

## Abstract

The exponential growth of scientific literature yields the need to support users to both effectively and efficiently analyze and understand the some body of research work. This exploratory process can be facilitated by providing graphical abstracts–a visual summary of a scientific publication. Accordingly, previous work recently presented an initial study on automatic identification of a central figure in a scientific publication, to be used as the publication’s visual summary. This study, however, have been limited only to a single (biomedical) domain. This is primarily because the current state-of-the-art relies on supervised machine learning, typically relying on the existence of large amounts of labeled data: the only existing annotated data set until now covered only the biomedical publications. In this work, we build a novel benchmark data set for visual summary identification from scientific publications, which consists of papers presented at conferences from several areas of computer science. We couple this contribution with a new self-supervised learning approach to learn a heuristic matching of in-text references to figures with figure captions. Our self-supervised pre-training, executed on a large unlabeled collection of publications, attenuates the need for large annotated data sets for visual summary identification and facilitates domain transfer for this task. We evaluate our self-supervised pretraining for visual summary identification on both the existing biomedical and our newly presented computer science data set. The experimental results suggest that the proposed method is able to outperform the previous state-of-the-art without any task-specific annotations.

## 1 Introduction

Finding, analyzing, and understanding scientific literature is an essential step in every research process, and one that is becoming ever-more time-consuming with the exponential growth of scientific publications (Bornmann and Mutz, 2015). To provide efficient means of analyzing the large body of research papers, researchers in natural language processing have focused on automatic summarization of scientific publications (e.g., Cohan et al., 2018; Cohan and Goharian, 2015; Mei and Zhai, 2008; Qazvinian and Radev, 2008; Lauscher et al., 2017; Yasunaga et al., 2019, *inter alia*). While most existing work on summarization of scientific publications focuses on the textual content of the publication only, in many research disciplines figures are an indispensable part of the paper, one that convey a wide range of information, e.g., about the data used in the study, the experimental design, or the empirical results. Furthermore, figures often convey information more effectively than text, since humans better remember and recall visual information (Nelson et al., 1976). For example, a deep neural network architecture is easily understandable as a figure or numerical values from experimental results can be easily compared in a bar chart or a plot.

Acknowledging the importance and usefulness of scientific figures for supporting users in their literature research, the publisher Elsevier recently started requesting authors to submit a visual summary of research called Graphical Abstract (GA), which is “a single, concise, pictorial and visual summary of the main findings of the article”^1^. GAs are displayed on the article page and also as part of the search results, so that the users are exposed to a more informative summary of a paper at a glance, even when the space for presentation is limited as is the case with search results. For example, the British Machine Vision Conference 2020 displayed a single figure on the paper browsing system^2^ along with paper title and author list so that participants could easily find relevant papers. In such settings, providing all paper figures is impractical and only a single, most representative figure needs to be selected as the visual summary of the work. Furthermore, GAs have also been shown to be beneficial to the authors themselves as they can improve their visibility (Oska et al., 2020).

While many authors do provide GAs as visual summaries of their research (i.e., they manually select the most representative figure), GAs are still not available for most publications (and especially so for older publications). To allow for the use of GAs in large-scale scenarios, Yang et al. (2019) proposed the novel task of identifying a central figure from scientific publications, i.e., selecting the best candidate figure that can serve as GA. In their work, they asked authors of publications uploaded to PubMed^3^ to select the most appropriate figure among all figures in their paper as the central figure. For 87.6% of the publications the authors clearly identified the central figure in their work, rendering the task of visual summary identification as well-defined. Based on the constructed data set, Yang et al. further proposed a method for central figure identification, which relies on supervised machine learning.

There are, however, two important limitations of this seminal work of in Yang et al. First, the proposed data set consists of PubMed papers only, limiting their experimental findings and results, i.e., the validation of the effectiveness of their approach to biomedical (i.e., life science) domains only. Lee et al. (2018) recently show that the use of figures varies drastically across different fields of research and, accordingly, central figure identification in other domains (e.g., computer science) may be substantially more or less challenging than in PubMed publications. It is therefore important to evaluate the effectiveness of visual summary identification methods across domains in order to assess their generality. The only existing data set, being tied to a single research area (i.e., biomedicine/life science), prevents such more comprehensive evaluations. In order to allow for a wider exploration of the central figure identification task, we need annotated data for at least one more domain, preferably with sub-domains. In this work, we introduce a new dataset for visual summary identification, covering four areas of computer science. Next, we acknowledge that the current approach of developing domain-specific models from annotated in-domain data is time-consuming and expensive, and can hardly be a viable solution for covering the wide variety of research areas and domains. While crowdsourcing can sometimes used to economically collect annotations for supervised machine learning tasks, this is unfortunately not the case for scientific publication mining. Domain knowledge is essential for understanding a scientific publication, and therefore only domain experts (e.g., university students or researchers in respective disciplines) can reliably annotate data for central figure identification. As a result, collecting training data for central figure identification for various research fields is impractical due to expert knowledge requirements for annotation on a scientific paper. We thus propose a more viable, transfer learning approach, based on a self-supervised learning objective.

**FIGURE 1:**
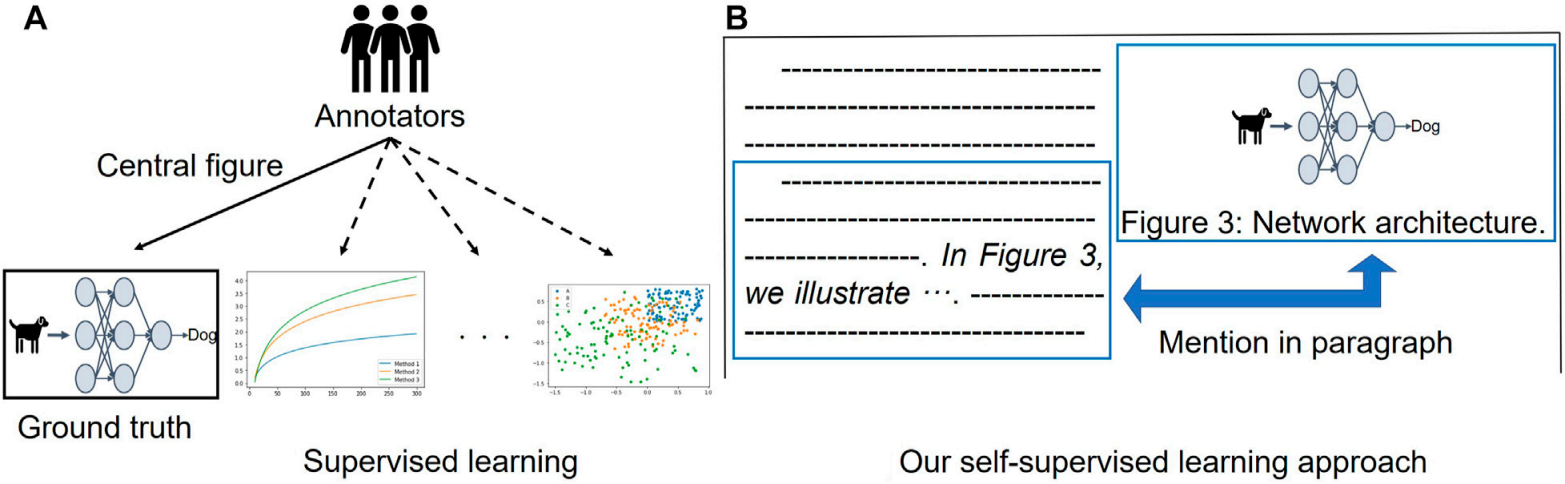
Illustration of our proposed self-supervised learning for central figure identification. **(A)** Existing supervised learning approach (Yang et al., 2019) requires labeled data for model training. **(B)** Our proposed self-supervised learning utilizes an inline reference to figure, which can be obtained without manual effort.

Based on our workshop paper (Yamamoto et al., 2021), we tackle the problems mentioned above by 1) building a novel benchmark for central figure identification, consisting of computer science (CS) papers from several CS subareas, and 2) proposing a novel self-supervised learning approach that does not require manually annotated data for central figure identification. To build our proposed benchmark for the task, we hire two (semi-expert) annotators to read a paper’s abstract and rank the top three figures to be the best candidates for the GA. We collect papers presented at several conferences from four CS subdomains: natural language processing (NLP), computer vision (CV), artificial intelligence (AI), and machine learning (ML). The novel data set enables us to evaluate the performance of models for central figure identification across different (sub)domains as well as to investigate robustness of the central figure identification models to domain transfer. Secondly, we introduce a self-supervised learning approach for central figure identification that removes the need for manual annotation for model training. In our approach, instead of employing a ground-truth label indicating the central figure, we exploit inline references to the figures (Figure 1): in the body of the article, a figure is usually mentioned with a direct reference like “*In* Figure 3, *we illustrate* ⋯ *”*, which typically indicates that the content of the mentioning paragraph (containing the inline reference) is relevant for what the mentioned figure illustrates. We create pairs of paragraphs in an article’s body and mentioned figures as training data. We then train a Transformer-based (Vaswani et al., 2017) model to predict a score that reflects whether a given paragraph from the article is connected to a caption of a paired figure. At inference time, we consider pairs of abstracts and figure captions as the model’s input to predict whether the content of the figure matches the article’s abstract (i.e., the overview of the article). This stands in contrast to sentence matching (Bowman et al., 2015; Wang et al., 2017; Duan et al., 2018; Liu et al., 2019), which is usually treated as a sentence-pair classification task: instead, we cast the problem as a ranking task according to how much the content of the figure caption matches the content of the article’s abstract. Without the use of any manually labeled data for training, our self-supervised approach outperforms the existing fully supervised learning approach (Yang et al., 2019) in terms of top-1 accuracy on the existing data set consisting of PubMed publications. Finally, we provide a comparison of central figure identification across training data from different domains.

## 2 Related Work

While the automatic creation of a textual summary from scientific paper has been widely studied (Cohan et al., 2018; Cohan and Goharian, 2015; Mei and Zhai, 2008; Qazvinian and Radev, 2008; Lauscher et al., 2017; Yasunaga et al., 2019), only a few studies have focused on the visual aspects of scientific publications. For a different form of a summary of a scientific paper, Qiang et al. (2016) proposed a method for automatic poster generation from scientific publications, where the output consists of texts and figures. However, in their approach, a manual selection of figures is required. Sun et al. (2021) presented a presentation slide generation system that retrieves the contents from a given paper and generates multiple slides with text and figures. In contrast to this system, which typically generates multiple pages including multiple figures, we focus on identifying central figures, i.e., standalone figures representing a visual summary of the publication. Liu and Yu (2014) and Yu et al. (2010) investigated approaches for figure ranking from a single paper based on their importance. Another problem which is centered around figures in scientific publications is keyword-based figure retrieval (Kuzi and Zhai, 2019). Kuzi and Zhai (2021) later investigated neural-network-based embeddings of figures in scientific publications.

In this paper, we study central figure identification, where the task is to identify the best candidates for the GA, a visual summary of a paper (Yang et al., 2019). Similar to extractive text summarization (Zhong et al., 2020; Cheng and Lapata, 2016; Xu and Durrett, 2019; Nallapati et al., 2017), which extracts important sentences from the original text, we consider the task as extracting a single figure which can serve as a central figure from a set of figures in a paper. Central figure identification is also related to multi-modal summarization (Zhu et al., 2018, 2020) in which both sentences and images are extracted from a document. Several studies have been conducted on GAs, including their use (Yoon and Chung, 2017), design pattern (Hullman and Bach, 2018), and effect (Oska et al., 2020). Closest to our work, a method for automatic central figure identification was first proposed by Yang et al. (2019). However, there exist two limitations in their work. First, they built an annotated data set for central figure identification, but include only papers from the biomedical and life science domains. This limits the wider applicability of methods trained on this data set, as the use of figures varies across different fields of study (Lee et al., 2018). To study central figure identification in a different domain, we propose a novel data set of computer science papers from several subdomains. The other limitation of Yang et al. (2019) is the use of a supervised machine learning algorithm, which requires a large amount of labeled data. Domain knowledge is necessary to annotate scientific publications, and therefore annotation on scientific publications is difficult for the non-expert (e.g., crowdsourcing workers). Indeed, existing data sets for various tasks in scientific publication mining (Lauscher et al., 2018; Hua et al., 2019; Yang et al., 2019; Yasunaga et al., 2019) are limited in terms of size, which additionally suggests that obtaining a sufficient number of data for supervised machine learning on scientific text is expensive and time-consuming. To remedy this bottleneck of annotation cost, we propose a self-supervised approach in which we use direct inline figure references in the article body to heuristically pair article paragraphs with figure captions and use those pairs as distant supervision.

Based on Yang et al. (2019)’s finding that the similarity between an abstract and a figure caption is most indicative for solving the task, we focus on finding the figure which reflects the content of the article’s abstract best. Accordingly, we regard central figure identification as a text-matching task, aiming to find the figure caption that matches an abstract’s text best. Generally, there are currently two popular types of text matching approaches: sentence encoding-based and attention-based. The sentence encoding approach obtains representations of two texts separately (Bowman et al., 2015; Reimers and Gurevych, 2019) and then relies on learning how to match these representations. In the attention-based approach, an attention mechanism is used, which captures the semantic interaction between two texts (Duan et al., 2018; Liu et al., 2019; Wang et al., 2017). In this paper, we propose an approach which falls into the second category and is based on pre-trained Transformers (Beltagy et al., 2019; Devlin et al., 2019).

## 3 Annotation Study

The work of Lee et al. (2018) indicates that the use of figures in a scientific publication is quite different among fields of study, but the only existing annotated data set is limited to PubMed papers (biomedical and life science domain) only (Yang et al., 2019). To study central figure identification in a different domain, we build a new data set consisting of computer science papers.

**Data Collection.** As opposed to many disciplines, conference proceedings are considered formal publications in computer science (Eckmann et al., 2012). We first select several conferences in computer science where their proceedings are available as open access. To compare central figure identification in various research domains, we collect papers published between 2017 and 2019 at top-tier conferences in four subdomains, namely natural language processing (NLP), computer vision (CV), artificial intelligence (AI), and machine learning (ML). Some papers in the existing data set (Yang et al., 2019) only include one or two figures, but we only keep papers with more than five papers to include more challenging instances in our data set. The statistics of the data set, including the number of publications and the average number of figures per publication for each subdomain, are summarized in Table 1.

**TABLE 1:** Statistics of our newly proposed data set for central figure identification in computer science.

Domain	NLP	CV	AI	ML	Total
Conferences	ACL	CVPR	AAAI	ICML	—
	EMNLP	—	IJCAI	—	—
No. papers	148	158	147	144	597
Two annotators	126	127	120	123	496
Single annotator	22	31	27	21	101
*Figures/paper*	—	—	—	—	—
Average	6.2 ± 1.8	7.0 ± 1.8	6.1 ± 1.5	6.5 ± 1.9	6.5 ± 1.8
Minimum	5	5	5	5	5
Maximum	13	13	14	13	14

**Annotation Process.** In our annotation task, annotators are asked *to rank the top 3 figures that reflect the content of the abstract best and can therefore be considered candidates for the GA of the paper*. We adopt the definition of a GA as provided in the Elsevier author guidelines (cf. footnote^1^). We hired two coders with a university degree in computer science, who were instructed to study the examples provided on the publisher page and discuss them in a group to make sure they understood the notion of a GA.

**FIGURE 2:**
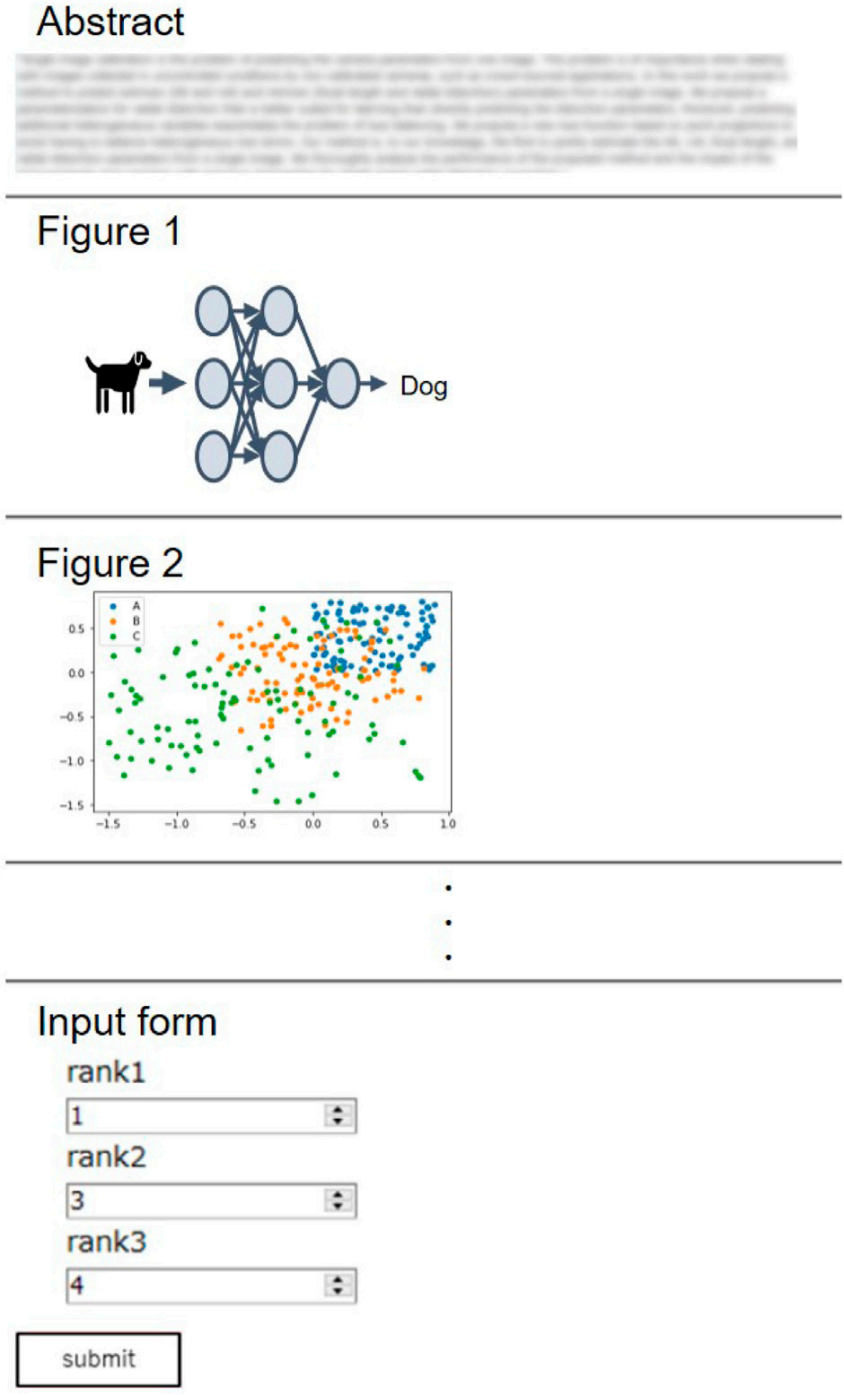
Graphical user interface for annotation. Paper abstract is displayed at the top to provide the overview of the research. Figures are then shown in randomly shuffled order. Annotators are asked to fill figure number of top three figures that reflect the content of abstract in the input form. The answer is recorded after clicking a “submit button”.

The annotation process was conducted with a web-based tool with a graphical user interface (Figure 2), which we developed for the purpose of our study. Annotators first read a paper abstract to grasp the overview of the research. All figures extracted from the same paper are displayed below the abstract, randomly shuffled to avoid bias due to the order. After reading the abstract, annotators are asked to rank the top 3 figures as potential candidates for the GA. The majority of instances have been annotated by both annotators, while a limited number of samples have been labeled by only one annotators, as summarized in Table 1. The inter-annotator agreement across the doubly annotated data amounts to 0.43 Krippendorff’s *α* (ordinal), which, while denoting fair agreement, also points to the difficulty and subjectivity of the task.

## 4 Methodology

We present our self-supervised approach for central figure identification.

**Problem Definition.**Yang et al. (2019) introduced two types of central figure identification problems, figure-level, and paper-level. In the figure-level setting, the task is cast as a classification problem where a given figure is classified as either a central figure or not. In the paper-level setting, a central figure is selected from the set of figures in a single paper. Our interest lies in summarizing a scientific publication with its visual content (i.e., figure) by means of identifying a single figure that would best serve as a visual summary of a paper. Instead of the simple binary classification approach, in which one would classify pairs of text and figures (captions) as matching or non-matching, being interested in ranking the figures by their suitability as the visual summary for the paper, we adopt contrastive learning approach in which force scores of positive pairs (i.e., figures that correspond to the inline text) to be scores higher than negative pairs (i.e., figures that do not correspond to inline text). Following the result that the similarity between an abstract and a figure caption is the most important factor for central figure identification (Yang et al., 2019), we focus on identifying a figure which best matches the content of the abstract. Concretely, we learn a scoring function *f* (*x*, *y*) that predicts the degree to which a figure *x*_*i*_ ∈ *X* matches the text content *y*. All figures are then ranked according to the model’s prediction *S* = {*s*_*i*_: *s*_*i*_ = *f* (*x*_*i*_, *y*)}. Instead of employing pairs consisting of an abstract and a figure caption, which corresponds to the final prediction goal but requires annotations, the model is trained with pairs of paragraphs with inline references to a figure and figure captions.

**FIGURE 3:**
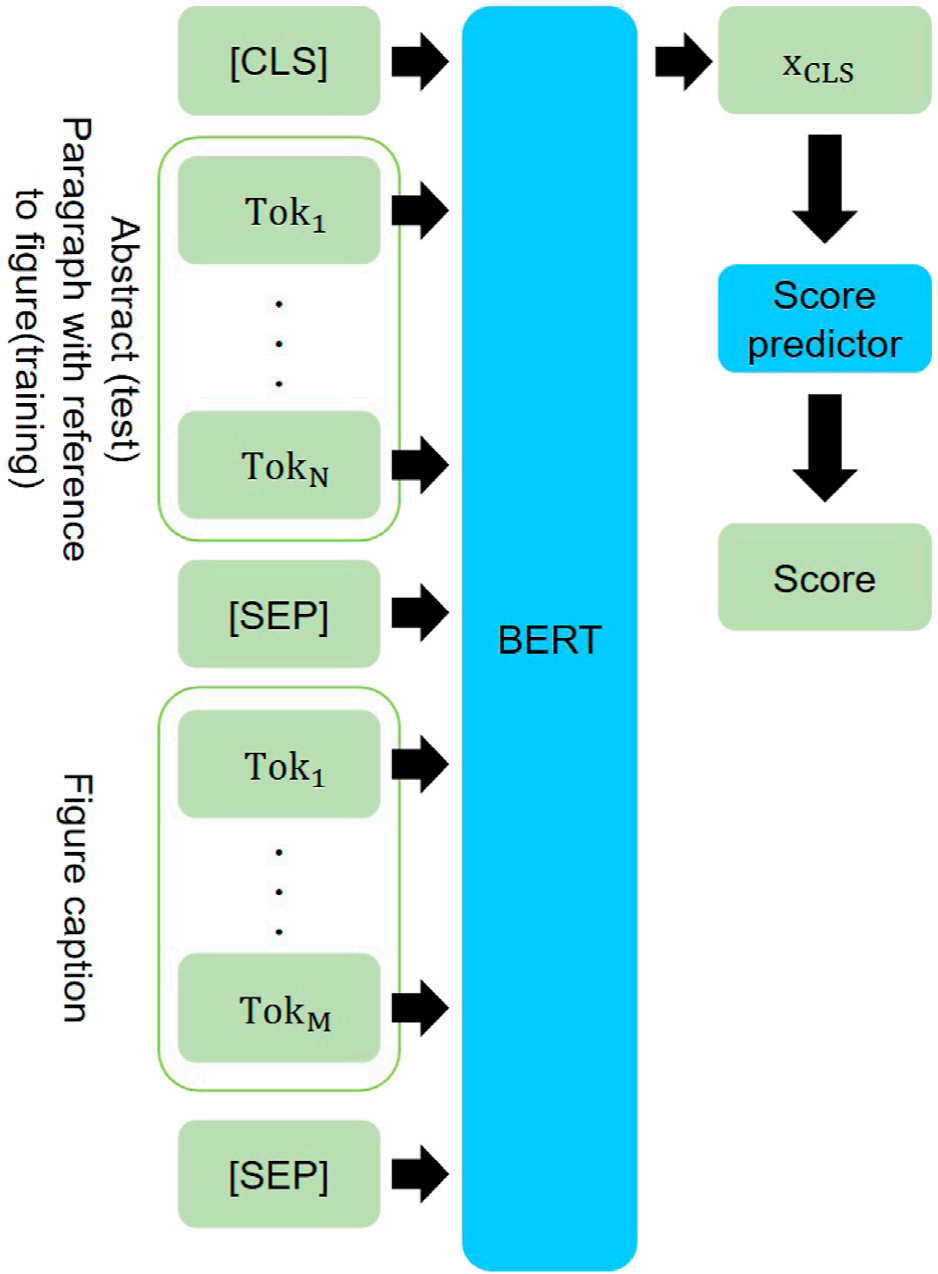
Our model for abstract-caption pair scoring. Paragraphs explicitly mentioning figures are paired with the figure captions during training.

**Model.** We build the model that predicts appropriateness of a figure to be selected as the central figure, given its caption and the abstract of the paper. The proposed model consists of a Transformer (Vaswani et al., 2017) encoder with a score prediction layer (Figure 3).

Inspired by the recent attention-based approach for sentence matching (Wang et al., 2017; Duan et al., 2018; Liu et al., 2019) and the success of pre-trained language model in NLP, we opt for pre-trained Transformers (Beltagy et al., 2019; Liu et al., 2019; Devlin et al., 2019) as the text encoder. As input to the Transformer encoder, a figure caption is paired with an abstract (inference) or the paragraph from the body of the article which explicitly mentions the figure (training). We further insert the Transformer’s special tokens as “(CLS) abstract/paragraph (SEP) caption (SEP).” The last hidden representation of the (CLS) token, **x**_*CLS*_, is then fed to a score prediction layer with a linear transformation that produces the final relevance score: *s* = **x**_*CLS*_ **W** + *b*, with the vector $W\in R^{H}$ and scalar b∈R as regressor’s parameters (*H* = 768 is BERT’s hidden state size). The sequence length of BERT is limited to up to a maximum of 512 tokens, which makes it difficult to feed an entire abstract. One possibility to overcome this obstacle is increasing the maximum sequence length, but we declined this option due to the requirement of training instances with longer sequences and huge GPU memory. To allow for abstracts of longer sequences, we divide an abstract into sentences and aggregate scores across sentences. Given a function *g* (*x*, *y*_*n*_) which scores pairs of a figure caption *x* and a sentence (in an abstract) *y*_*n*_ ∈ *Y* where *Y* = {*y*_*n*_: *n*} is a set of sentences in an abstract, the scoring function is defined as *f* (*x*, *y*) = *∑*_*n*_ *g* (*x*, *y*_*n*_).

**FIGURE 4:**
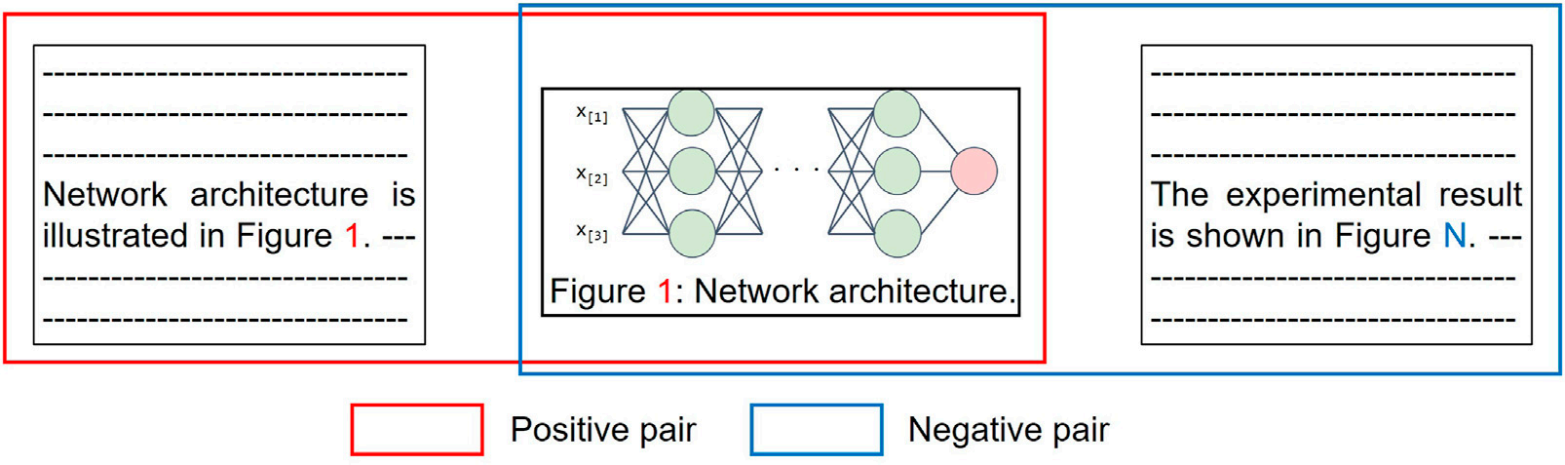
Creation of paragraph-figure pairs used as training instances for our models. For a single training instance, positive and negative pairs are created. A figure is paired with paragraphs which refers to the figure and different one in positive and negative pairs, respectively.

**Training Instance Creation.** Whereas supervised machine learning requires a large amount of training data, annotating on scientific publications is expensive and time-consuming as they are highly technical texts. To overcome the difficulty in collecting training data, we introduce a self-supervised approach by leveraging explicit inline references to figures (e.g., “Figure 2 *depicts the results of the ablation experiments …* ”), which does not require any manual effort (Figure 4). In a scientific publication, an inline reference to a figure suggests a connection between the paragraph and the figure. We denote the set of paragraphs that mention figures as $D=\left\{d_{i}^{j}:i\right\}$ where $d_{i}^{j}$ is a *i*-th paragraph that mentions the figure *x*_*j*_. During model training, we learn the matching problem of the figure *x* and the paragraph *d*, which results in the ability to match the text and the figure. As training data, we create positive and negative pairs where paragraphs are paired with referred and non-referred figures, respectively, i.e., we treat a pair $\left(x_{i},d_{j}^{k}\right)$ as positive training instance if *i* = *k* and as negative if *i* ≠ *k*.

**Optimization.** Our training objective is ranking the positive pairs higher than negative ones. To avoid exceeding BERT’s maximum sequence length, we randomly sample a single sentence from a paragraph and couple it with a figure caption to form an input sequence as follows: “(CLS) sentence (SEP) caption (SEP).” Similar to the Triplet loss (Hoffer and Ailon, 2015), we optimize the following loss function: $L=\max\left(s_{p}-s_{n}+\alpha ,0\right)$, where *α* is set to *α* = 1.0 and *s*_*p*_ and *s*_*n*_ denote the scores for the positive and negative pairs, respectively. For a single training instance, one positive and one negative pair which deal with the same figure *x*_*i*_ are sampled as $\left(x_{i},d_{j}^{i}\right)$ and $\left(x_{i},d_{k}^{i^{\prime }}\right)\;\left(i\neq i^{\prime }\right)$, respectively. The model is optimized to predict that the score for a positive pair is lower than that for a negative one, and therefore, the figure with the lower score is considered as more suitable for a central figure at test time.

## 5 Experiments

We present our experiments on biomedical and life-sciences as well as on computer science publications. We evaluate proposed self-supervised learning with a BERT-based model.

### 5.1 Implementation Details

The experimental code is implemented using the BERT implementation of the Hugging Face library (Wolf et al., 2019). We employ the Adam optimizer (Kingma and Ba, 2014) with the learning rate 1*e—*6, batches of size 32, dropout at the rate of 0.2, and a gradient clipping threshold of 5. We train the model for one epoch with four NVIDIA V100 GPUs. We run the experiment with five different random seeds and report the average value of each evaluation metric.

The PDF versions of papers are collected as a source of text and figures. We use the Science Parse library^4^ to obtain the body text of an article. The extracted text’s explicit inline references are identified via the keywords “Figure” or “Fig.” Figure captions are extracted using the image-based algorithm proposed by Siegel et al. (2018). We mask the figure number (in both the inline mention and figure caption), in order to prevent the model from overfitting to figure numbers (i.e., as any undesirable bias/skewness in figure number distributions in our training data could reduce the model’s generalizability). For reproducibility, we release our sample implementation. (https://github.com/yamashin42/Visual-Summary).

### 5.2 Dataset

We conduct the experiments on both the existing PubMed data set (Yang et al., 2019) and our newly proposed computer science data set.

**PubMed.**Yang et al. (2019) proposed a data set of 7, 295 biomedical and life science papers from PubMed for the problem of central figure identification. One of the figures from a single paper is labeled as a central figure by the authors of each publication. We downloaded the 7, 113 publicly available PDFs of articles from PubMed and used training, validation, and test split provided by Yang et al. in the ratio of 8:1:1. We create 40*k* paragraph-figure pairs from the training set using our figure mention heuristic and use these 40*k* samples for model training. Following Yang et al., we evaluate the top-1 and top-3 accuracy performance as only single figure is labeled as a central figure per paper.

**Computer Science (CS).** We also evaluate our proposed approach on our proposed data set of CS papers (Section 3). We use all labeled papers annotated by both two and single annotator(s) for evaluation. As opposed to the PubMed data set, in which only a single figure is labeled, top-3 figures are annotated per paper in our data set. We therefore evaluate the performance of central figure identification on three ranking metrics: Mean Average Precision (MAP), Mean Reciprocal Rank (MRR), and normalized Discounted Cumulative Gain (nDCG). We collect papers from the same subdomains as the annotated test data for the training instances, presented between 2015 and 2018, and divided them into training and validation in the ratio of 9:1. From the training portion, we create 40*k* paragraph pairs and use all 40*k* samples for model training.

### 5.3 Experimental Result

**Performance on the PubMed data set.** We first experiment on the PubMed data set (Table 2). As baselines, we provide the results of the following two ranking methods from (Yang et al., 2019):

**TABLE 2:** Performance of vanilla BERT, RoBERTa and SciBERT on the PubMed data set (Yang et al., 2019). We report Accuracy@1 and Accuracy@3.

Method	Model	Accuracy@1	Accuracy@3
Baseline	Random	0.280	0.701
	Pick first	0.301	0.733
Yang et al. (2019)	Text-only	0.333	**0.810**
	Full	0.344	0.793
Ours	Vanilla BERT	0.331	0.770
	RoBERTa	0.347	0.741
	SciBERT	**0.383**	0.787

• Random: figures from a single paper are randomly ranked;

• Pick first: figures from a single paper are ranked as the order of appearance (e.g., Figure 1 is first, figure N is *N*th).

We also compare with the existing supervised machine learning approach (Yang et al., 2019). Following the results reported by Yang et al., logistic regression is used as the underlying machine learning algorithm. We report results for two variants of their methods:

• Text-only: the cosine similarity of the TF-IDF representation between the paper’s abstract and figure caption is used as an input feature;

• Full: in addition to the text-only setting, the figure type label (e.g., diagram, plot) and layout (e.g., section index, figure order) are provided as input features.

For our proposed model, we evaluate three variants of pre-trained Transformers, namely vanilla BERT (Devlin et al., 2019), RoBERTa (Liu et al., 2019), and SciBERT (Beltagy et al., 2019).

The experimental result shows that all variants of pre-trained Transformer encoders outperform the baselines in terms of both top-1 and top-3 accuracy. SciBERT is the best among the text encoders because of the in-domain pre-training. Moreover, the SciBERT model also outperforms the existing supervised learning method (Yang et al., 2019) in terms of top-1 accuracy. The experimental result indicates that the model obtained the ability to identify the figure that reflects the content of an abstract without seeing an actual abstract-caption pair during training. As the model cannot learn from an abstract, which is only given as an input at inference time, we can expect to improve the performance further using domain adaptation techniques (Ramponi and Plank, 2020) to account for the domain-shift between abstract and paragraph from the article body.

**Performance on the CS data set.** We also evaluate the performance of the model on our CS data set (Table 3). Following the experiment on the PubMed data set, we provide the evaluation of two baselines (random and pick first) for comparison. Here, we compare three variants of pre-trained Transformers, vanilla BERT, RoBERTa, and SciBERT, to verify the effectiveness of SciBERT in the CS domain since the majority of samples in the corpus for SciBERT pre-training is from the biomedical domain and the only 18% is from CS domain.

**TABLE 3:** Performances of vanilla BERT, RoBERTa, and SciBERT against the random and pick first baselines on CS papers. We report Mean Average Precision (MAP), Mean Reciprocal Rank (MRR) and Normalized Discounted Cumulative Gain (nDCG). Best performances are highlighted in bold.

Method	Model	MAP	MRR	nDCG
Baseline	Random	0.616	0.693	0.732
	Pick first	**0.754**	**0.827**	**0.809**
Ours	Vanilla BERT	0.694	0.773	0.767
	RoBERTa	0.702	0.793	0.775
	SciBERT	**0.731**	**0.822**	**0.794**

Our self-supervised learning approach outperforms the random baseline in terms of MAP, MRR, and nDCG, which indicates that our approach can gain the ability to identify a central figure in the CS domain as well as biomedical science domain. As with the case of PubMed papers, SciBERT performs the best in CS papers among the different variants of pre-trained Transformers. Though most data used for SciBERT pre-training is from papers in the biomedical domain, a certain number of CS papers seen in pre-training still contribute to the downstream performance on central figure identification.

**FIGURE 5:**
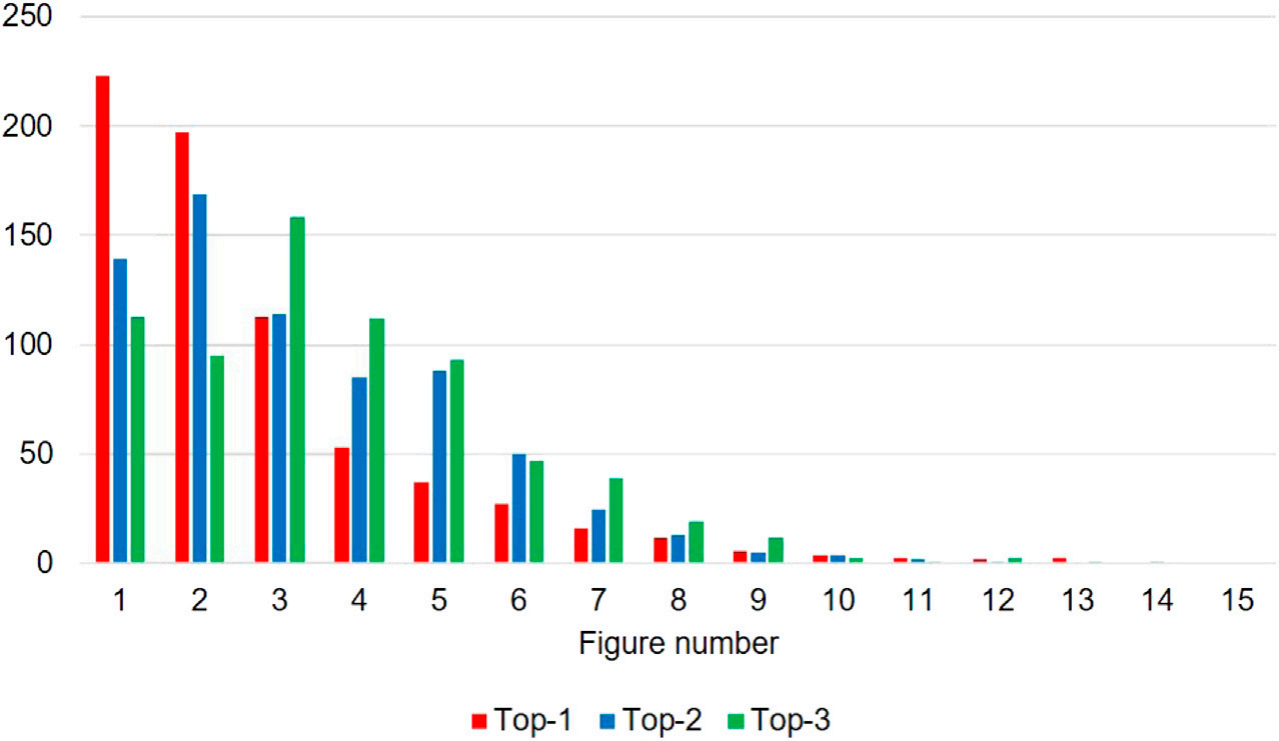
Histogram of figure numbers annotated as top-3 figure in our proposed CS data set.

However, in contrast to the case of PubMed papers, the “pick first” baseline outperforms our Transformer-based approach. This result means that the annotators tend to select figures that appear at the earlier part of a paper as top-3 figures. We then visualize the histogram of figure number to verify the annotation is biased by the order in which the figures appear (Figure 5). Whereas figures are randomly shuffled in our annotation system, and therefore annotator did not know the figure number, earlier figures tend to be ranked higher. Note again that the annotated papers contain at least five figures. For example, Figure 1 is selected as rank one in 32.0*%* of annotated samples. A similar phenomenon has been observed in text summarization on the news domain: important information tends to appear in the earlier part of the article (Kryscinski et al., 2019). See et al. (2017) then exploited such bias and found that using the first 400 tokens shows better performance than using the first tokens in text summarization of news articles. Whereas Yang et al. (2019) report that using only the first figures degrades the performance in central figure identification on the PubMed data set, our findings indicate that exploiting the bias caused by the order of the figures may be beneficial in other domains, e.g., CS. We consider to explore this path in future work.

**Cross-domain Comparison.** Prior work of Lee et al. (2018) revealed that the use of figures in scientific publications is different across different fields of study. The fact arises the question of whether this is the case with central figure identification. We then conduct an experiment to evaluate the robustness of our approach across research domains. We train the model on the papers of one of the domains and compare the performance across various domains: for example, the model is trained on PubMed data set and evaluated on CS data set. We consider two settings: biomedical vs computer science and across different CS subdomains (NLP, CV, AI, and ML).

We first compare the performance of models trained on biomedical publications from the PubMed data set and CS publications from our new data set (Table 4). These two domains are quite different research fields, and accordingly, papers from these domains are written in a different way, e.g., the terminology differs.

**TABLE 4:** Performances of our vanilla BERT and SciBERT models trained on the PubMed and CS data set evaluated on the PubMed and CS data set compared against the random baseline. For PubMed, we report Accuracy@1 (Acc@1) and Accuracy@3 (Acc@3), for CS we report Mean Average Precision (MAP), Mean Reciprocal Rank (MRR) and Normalized Discounted Cumulative Gain (nDCG). We indicate the best model performances in bold, and the in-domain setup in light blue.

Model	Training Data	Pubmed		CS		
		Acc@1	Acc@3	MAP	MRR	nDCG
Random	–	0.280	0.701	0.616	0.693	0.732
Vanilla BERT	PubMed	0.331	0.770	0.662	0.751	0.772
	CS	0.307	0.750	0.694	0.773	0.767
SciBERT	PubMed	**0.383**	**0.787**	0.728	**0.822**	0.789
	CS	0.368	0.777	**0.731**	**0.822**	**0.794**

In the case of SciBERT-based models, training on the PubMed papers results in better performance in terms of top-1 and top-3 accuracy on the PubMed data set. Similarly, training on the same domain as the test data yields the best performances in terms of MAP and nDCG on the CS data set. However, training on the papers of different domains, somewhat surprisingly, does not degrade the performance on both domains. We also examine the effects when using vanilla BERT, which is not pre-trained scientific text. Interestingly, we observe a larger gap in performance of the BERT models when fine-tuned either in the in-domain or cross-domain setup than with SciBERT (except for the case of nDCG in CS domain). We hypothesize that the samples of the target domain seen during pre-training contribute to the performance of the downstream task even without samples of the target domain in fine-tuning. Still, even the vanilla BERT model trained in the cross-domain setup outperforms the random baseline. This implies that papers from different domains exhibit roughly similar text-figure (caption) matching properties.

Next, we further examine the domain transfer with finer granularity, namely, we compare models trained on four subdomains of computer science (NLP, CV, AI, and ML). There are similarities and differences among these areas; for example, both studies in NLP and CV often utilize machine learning algorithms like deep neural networks, but CV papers typically contain more images than NLP publications. We train SciBERT-based models on four areas (NLP, CV, AI, and ML) and evaluate the performance on all domains (Table 5).

**TABLE 5:** Performances of our baselines (random, pick first) against SciBERT models trained on different CS domains (NLP, CV, AI, ML) evaluated on CS domains. We report Mean Average Precision (MAP), Mean Reciprocal Rank (MRR) and Normalized Discounted Cumulative Gain (nDCG). We indicate the best baseline and model performances in bold, and the in-domain setup in light blue.

	NLP			CV			AI			ML		
Model	MAP	MRR	nDCG	MAP	MRR	nDCG	MAP	MRR	nDCG	MAP	MRR	nDCG
Random	0.631	0.705	0.743	0.585	0.664	0.708	0.637	0.711	0.763	0.617	0.686	0.745
Pick first	**0.751**	**0.816**	**0.817**	**0.758**	0.831	**0.803**	**0.776**	**0.847**	**0.828**	**0.732**	**0.814**	**0.791**
NLP	0.727	0.791	0.777	0.716	0.826	0.785	0.727	0.828	0.799	0.676	0.759	0.762
CV	**0.728**	0.795	0.778	**0.721**	**0.833**	**0.790**	0.729	**0.834**	**0.802**	**0.682**	**0.769**	**0.763**
AI	**0.728**	0.795	0.776	0.716	0.826	0.785	0.728	0.830	0.800	0.679	0.763	0.760
ML	0.730	**0.798**	**0.779**	0.719	0.831	0.787	**0.730**	0.828	**0.802**	0.681	**0.769**	0.761

Overall, the performances are rather consistent, even training on the papers from different subdomains, which indicates that papers of the other topics can be used as training samples within computer science. Across the four topics, the performance gap in random and pick first baselines is the largest in the CV paper. As the papers from the CV domain contain more figures than those from the other fields (Table 1), randomly selecting a figure naturally results in worse performance. Another notable result is that the performance is the lowest on ML papers in all metrics. This indicates that central figure identification for ML is more difficult than for other CS domains, and accordingly, that the difficulty varies across the field of study, even within the computer science domain.

We then manually analyze instances from our CS data set to understand the difference across subdomains further. Among the figures ranked as top-1 by the SciBERT-based model in CV papers, 60.8*%* of figures illustrate a method, e.g., a neural network architecture. In 22.8*%* of the CV papers, an image is ranked as the highest figure by the SciBERT-based model. As research in CV focuses on understanding images, this type of figure is helpful in CV papers, for example, to describe the task or show the experimental result. Next, we manually analyze CV papers for which both the model prediction and the pick first baseline show high performance scores. In these papers, we find that certain types of figures are ranked higher by the annotators: for instance, common types include visualizations of an overall concept or of the main idea. We also observe that these figures tend to be shown in the earlier part of the article (typically as the first or second figure), which is consistent with the result that pick first baseline is stronger in CV papers.

Next, we analyze ML papers given that the models exhibited lower performances on this domain than in the other three subdomains. In 41.0*%* of ML papers, the figures illustrating proposed methods are selected as a central figure by the model, which is a lower fraction than for CV papers. Similarly, fewer images are ranked first (9.0*%*) as only a limited number of ML papers focus on visual information. In contrast, figures which show numerical visualizations (e.g., bar chart, line chart) are the top candidates in 41.6*%* of the ML papers, while these types of figures are selected in only 9.0*%* of the CV papers. Accordingly, the model prediction differs between CV and ML papers. Additionally, we manually check some samples of ML papers for which the model performance is lower than the average performance. In these ML papers, we find that most (or even all in a certain number of papers) figures are used for a similar purpose. For instance, a paper may have a line graph and a bar graph to show an experimental result. Identifying the best candidate in these cases, where figures are rather similar is a difficult task, even for a human. Indeed, we notice differences in terms of ranks provided between two coders in such papers. Moreover, in some of the ML papers where figures tend to be similar, no figure depicting the overall concept or showing an overview of a framework (as common in CV papers) exists. Although Yang et al. (2019) reported that in their initial study, in 87.6% of the publications the authors were able to identify a central figure, the case analysis of ML suggests the existence of more cases in which identifying a central figure is difficult. The differences between results in the CV and ML domains emphasizes the need for more comprehensive evaluation setups, encompassing multiple domains and subdomain, in order to more reliably assess the robustness of the models.

**FIGURE 6:**
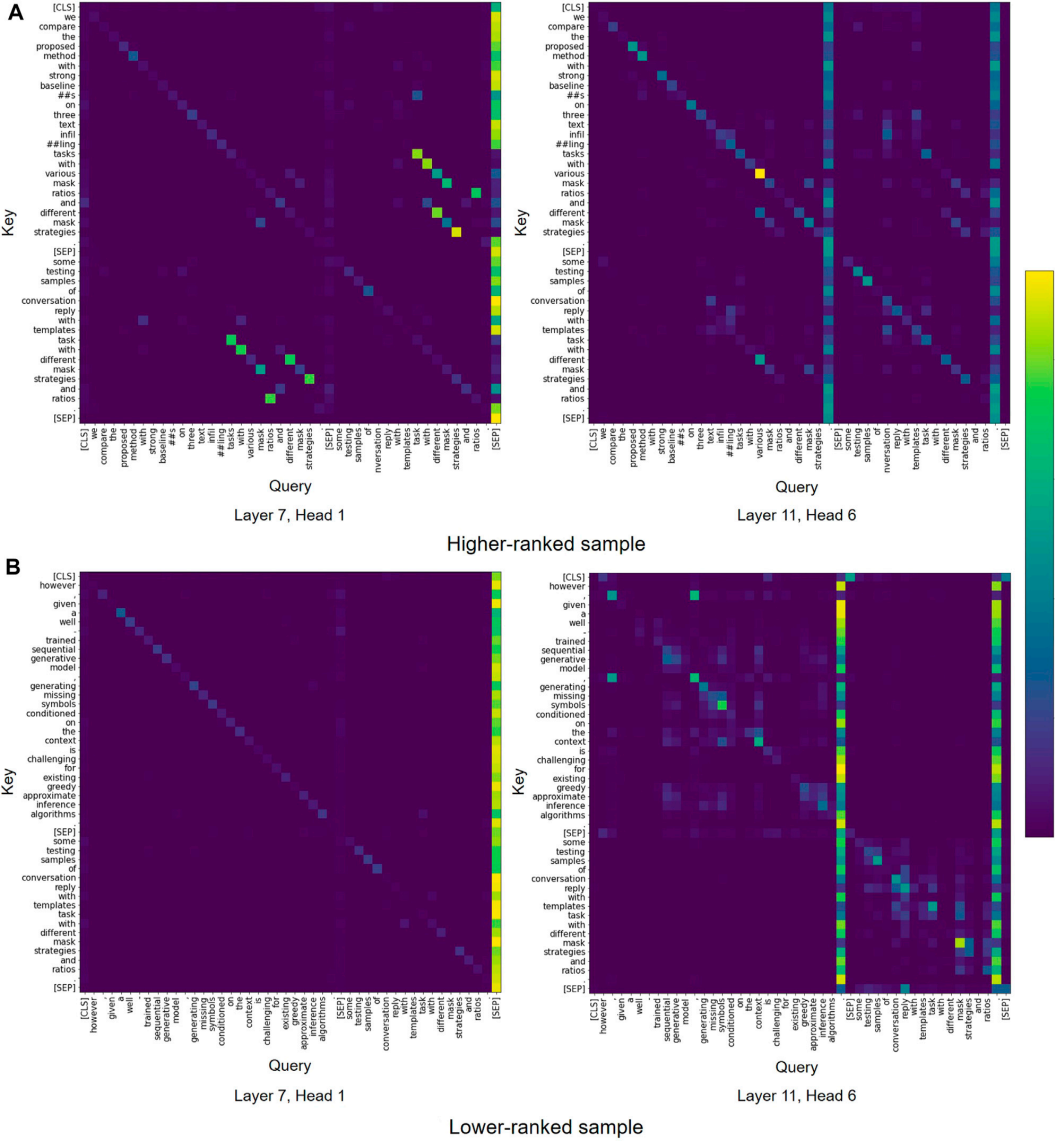
Examples of attention patterns in SciBERT model for **(A)** higher-ranked sample and **(B)** lower-ranked sample.

**Model Analysis.** To understand the model behavior, we analyze the attention patterns in Transformer models. We then visualize the attention maps from SciBERT to compare the samples that are ranked higher and lower by the model. From the visualization, we observe that most attention patterns are similar to those reported by Kovaleva et al. (2019), including vertical blocks, and heterogeneous blocks. As shown in Figure 6, we find some heads in SciBERT focusing on the lexical overlap between abstract and caption. In the example of the higher ranked sample (Figure 6A), some tokens like “mask” and “strategies” are used both in abstract and caption and have mutually high attention weights. Additionally, tokens “different” and “various” are used in similar meanings and show high attention weights. In contrast, in the lower ranked sample (Figure 6B), abstract and caption do not share semantically similar tokens except preposition ’with’ and therefore we cannot find the interaction between two sentences. This observation suggests that the lexical overlap between abstract and caption could be a basis for model’s judgement.

**FIGURE 7:**
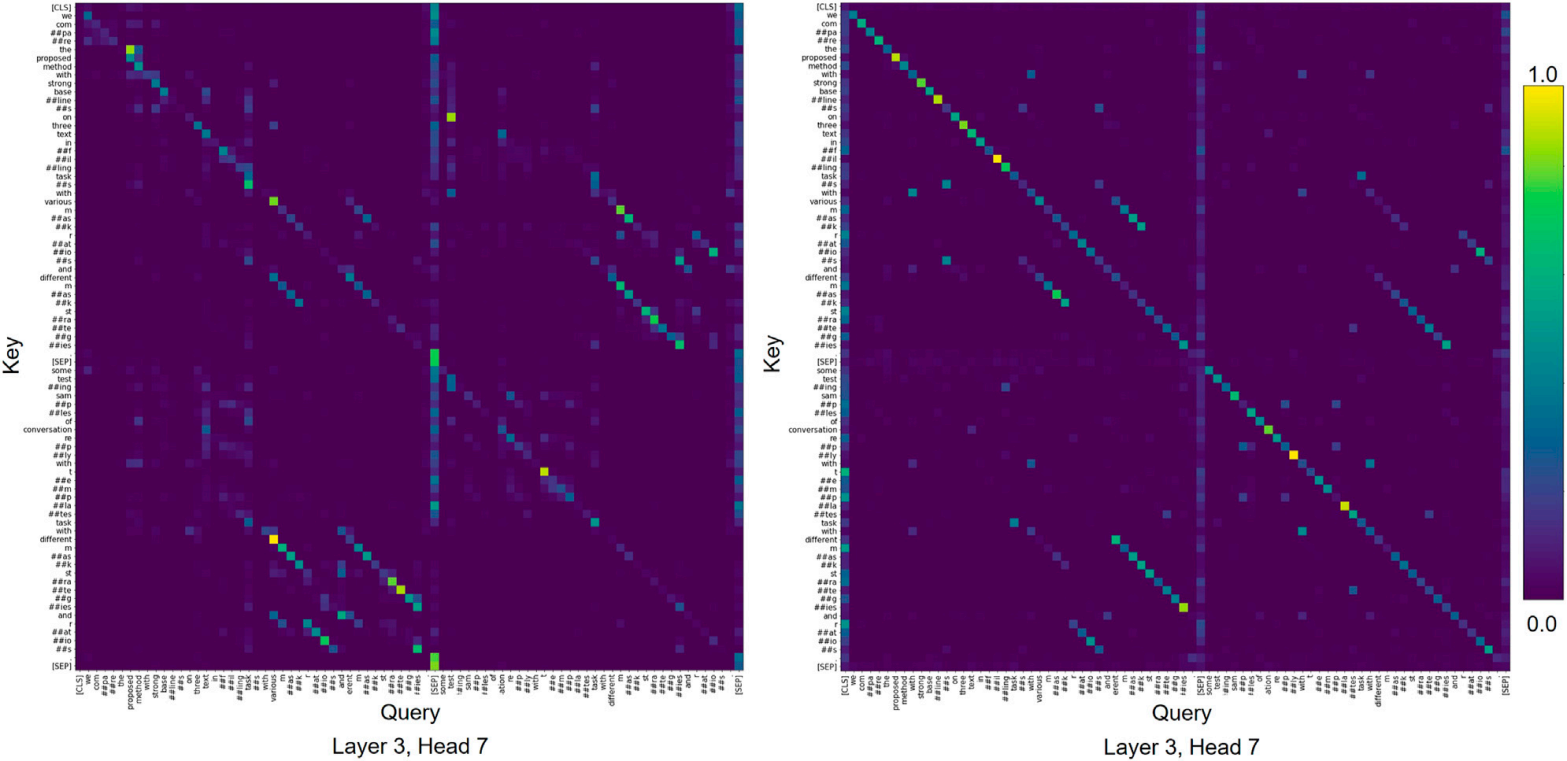
Examples of attention patterns in vanilla BERT model for higher-ranked sample.

We also find similar attention patterns in the vanilla BERT model, which is not pre-trained on the corpus of scientific text (Figure 7). Consequently, we conclude that the vanilla BERT model is able capture similar interactions between abstract and caption as observed with SciBERT.

Next, we quantitatively analyze the attention in the Transformer models. Here, we compare the attention patterns of the models trained on four subdomains of computer science (NLP, CV, AI, and ML). We adopt the cosine similarity as the metrics of similarity of attention maps as the attention analysis by Kovaleva et al. (2019). We randomly sample 100 sentence-caption pairs from CS papers and calculate the cosine similarity of flattened attention maps among the models (Table 6). All combinations of subdomains show high cosine similarity, indicating that attention patterns of SciBERT are consistent after fine-tuning on papers from different fields of CS. This observation supports the result that there is no performance gap between the models trained on papers of different CS subdomains (see Table 5).

**TABLE 6:** Comparison of attention maps from SciBERT models fine-tuned on different CS subdomains. Cosine similarity is calculated by flattening attention maps from all layers into a single vector.

	NLP	CV	AI
CV	0.9997	0.9998	0.9998
AI	0.9998	0.9998	-
ML	0.9997	-	-

According to Kovaleva et al. (2019), attention maps change the most in the last two layers of pre-trained BERT after fine-tuning. We then analyze the effect of task-specific fine-tuning on the attention patterns of SciBERT. We compare the two training settings: the standard fine-tuning, in which all SciBERT’s parameters are updated (the same as all our previous experiments), and feature-based training, in which all SciBERT’s parameters are fixed and only the regressor’s parameters are updated during training. The comparison of the performance and the attention maps are summarized in Table 7. The performance evaluation indicates that freezing SciBERT’s parameters results in performance degradation, which suggests the effect of fine-tuning. On the other hand, we also observe the high cosine similarity of attention maps between the fine-tuned and frozen SciBERT models. This suggests that only a slight update in the pre-trained Transformer have the potential to substantially change the predictions of the regressor.

**TABLE 7:** Evaluation of the effect of fine-tuning of SciBERT model on CS papers.

(a) Performance comparison on CS data set.			
SciBERT	MAP	MRR	nDCG
fine-tune	**0.733**	**0.827**	**0.794**
Freeze	0.677	0.752	0.754

(b) Cosine similarity of attention maps in each layer of fine-tuned and frozen SciBERT on randomly sampled 100 sentence-caption pairs from CS papers.						
Layer	1	2	3	4	5	6
Cosine similarity	0.9999	0.9993	0.9983	0.9982	0.9983	0.9980
Layer	7	8	9	10	11	12
Cosine similarity	0.9977	0.9971	0.9964	0.9951	0.9948	0.9945

## 6 Conclusion

In this work, we investigated the problem of central figure identification, the task to identify candidate figures that can serve as visual summaries of their scientific publication, referred to as Graphical Abstract (GA). Existing work (Yang et al., 2019) presented an annotated data set consisting of biomedical publications for the problem and proposed a method based on supervised machine learning. Annotating scientific publications requires expert knowledge of the domain and collecting large-scale annotated data for new domains is costly and time-consuming. Consequently, preceding research is limited to the central figure identification in the biomedical domain only. To alleviate these issues, we first presented a novel benchmark data set consisting of computer science papers presented at several conferences in four fields, including NLP, CV, AI, and ML. Moreover, we also proposed a self-supervised learning approach that only requires collecting scientific publications without manually annotating any data. The main intuition behind our approach is that an explicit inline reference to the figure indicates a semantic link between the content of the paragraph from the body of the article and the figure. We then train the model on the paragraph-caption matching problem and, at inference time, we consider central figure identification as abstract-caption matching task. Our experimental results show that our self-supervised learning approach is effective for central figure identification without any need for manually annotating data and outperforms the existing supervised approach in terms of top-1 accuracy. A deeper analysis across the different research domains indicated that model performances and attention patterns stay roughly consistent across the subdomains. However, interestingly, a qualitative analysis revealed that different types of figures are ranked higher in different subdomains; for example, general visualizations or overviews of methods tend to be ranked higher on CV papers, while figures visualizing numerical content (e.g., line graph, bar graph) are ranked higher on ML papers. This observation emphasizes the necessity for more datasets like the one we introduced in this work, in order to enable comparison of models’ central figure identification performance across different domains and subdomains. We hope that our study fuels further developments in automatic visual summary creation to provide more efficient and effective information access.

In our future work, we will explore the effect of larger-scale pretraining encompassing publications from a wider variety of research areas. As our pretraining is self-supervised, i.e., it does not require any manual annotations, we can scale it up by adding virtually any collection of scientific papers containing figures. We plan to investigate to which extent training on large-scale corpus like S2ORC (Lo et al., 2020) improves the model performance, and explore the tradeoff between the size of pretraining (time and compute) and actual performance on central figure identification.

## Data Availability

The raw data supporting the conclusions of this article will be made available by the authors, without undue reservation.

## Footnotes

1
https://www.elsevier.com/authors/journal-authors/graphical-abstract

2
https://www.bmvc2020-conference.com/conference/papers/?filter=keywords

3
https://pubmed.ncbi.nlm.nih.gov/

4
https://github.com/allenai/science-parse
